# Extended-Spectrum Beta-Lactamase Production and Carbapenem Resistance in Elderly Urinary Tract Infection Patients: A Multicenter Retrospective Study from Turkey

**DOI:** 10.3390/antibiotics14070719

**Published:** 2025-07-17

**Authors:** Çiğdem Yıldırım, Sema Sarı, Ayşe Merve Parmaksızoğlu Aydın, Aysin Kilinç Toker, Ayşe Turunç Özdemir, Esra Erdem Kıvrak, Sinan Mermer, Hasip Kahraman, Orçun Soysal, Hasan Çağrı Yıldırım, Meltem Isikgoz Tasbakan

**Affiliations:** 1 Department of Infectious Diseases and Clinical Microbiology, Niğde Training and Research Hospital, Niğde 51200, Turkey; orcunsysl@gmail.com; 2 Department of Intensive Care, Niğde Training and Research Hospital, Niğde 51200, Turkey; semadmc@gmail.com; 3 Department of Infectious Diseases and Clinical Microbiology, Ege University Medical School, İzmir 35100, Turkey; ayse.merve.parmaksizoglu@ege.edu.tr (A.M.P.A.); tasbakan@yahoo.com (M.I.T.); 4 Department of Infectious Diseases and Clinical Microbiology, Kayseri State Hospital, Kayseri 38039, Turkey; dr.aysin@gmail.com (A.K.T.); drayseturunc@gmail.com (A.T.Ö.); 5 Department of Infectious Diseases and Clinical Microbiology, Manisa Celal Bayar University Medical School, Manisa 45030, Turkey; esrakivrak@gmail.com; 6 Department of Infectious Diseases and Clinical Microbiology, Izmir Economy University Medical School, İzmir 35100, Turkey; sinan.mermer@hotmail.com; 7 Department of Infectious Diseases and Clinical Microbiology, Eskişehir Osmangazi University Medical School, Eskişehir 26040, Turkey; hasipkahraman@gmail.com; 8 Department of Medical Oncology, Ege University Medical School, İzmir 35100, Turkey

**Keywords:** antibiotic resistance, carbapenem resistance, elderly patients, ESBL production, Gram-negative bacteria, urinary tract infection

## Abstract

**Introduction:** Urinary tract infections (UTIs) remain a significant public health issue worldwide, particularly affecting the geriatric population with increased morbidity and mortality. Aging-related immune changes, comorbidities, and urogenital abnormalities contribute to the higher incidence and complexity of UTIs in elderly patients. Antimicrobial resistance, especially extended-spectrum beta-lactamase (ESBL) production and carbapenem resistance, poses a major challenge in managing UTIs in this group. **Methods:** This retrospective, multicenter study included 776 patients aged 65 and older, hospitalized with a diagnosis of urinary tract infection between January 2019 and August 2024. Clinical, laboratory, and microbiological data were collected and analyzed. Urine samples were obtained under sterile conditions and pathogens identified using conventional and automated systems. Antibiotic susceptibility testing was performed according to CLSI standards. Logistic regression analyses were conducted to identify factors associated with ESBL production, carbapenem resistance, and mortality. **Results:** Among the patients, the median age was 78.9 years, with 45.5% female. ESBL production was detected in 56.8% of *E. coli* isolates and carbapenem resistance in 1.2%. *Klebsiella* species exhibited higher carbapenem resistance (37.8%). Independent predictors of ESBL production included the presence of urogenital cancer and antibiotic use within the past three months. Carbapenem resistance was associated with recent hospitalization, absence of kidney stones, and infection with *non-E. coli* pathogens. Mortality was independently associated with intensive care admission at presentation, altered mental status, Gram-positive infections, and comorbidities such as chronic obstructive pulmonary disease and urinary incontinence. **Discussion:** Our findings suggest that urinary pathogens and resistance patterns in elderly patients are similar to those in younger adults reported in the literature, highlighting the need for age-specific awareness in empiric therapy. The identification of risk factors for multidrug-resistant organisms emphasizes the importance of targeted antibiotic stewardship, especially in high-risk geriatric populations. Multicenter data contribute to regional understanding of resistance trends, aiding clinicians in optimizing management strategies for elderly patients with UTIs. **Conclusions:** This study highlights that *E. coli* and *Klebsiella* species are the primary causes of UTIs in the elderly, with resistance patterns similar to those seen in younger adults. The findings also contribute important data on risk factors for ESBL production and carbapenem resistance, supported by a robust patient sample.

## 1. Introduction

Urinary tract infections (UTIs) are common health problems among older adults and can lead to significant morbidity and mortality [1]. The aging process is associated with a decline in immune response, reduced organ function, and an increase in comorbidities, all of which contribute to a heightened susceptibility to infections in geriatric patients [1]. In elderly individuals, UTIs often present with atypical symptoms. Rather than the classical signs, confusion, fatigue, deterioration in general condition, and occasionally fever are more prominent manifestations [2]. This atypical presentation can delay accurate diagnosis and treatment, thereby complicating the management process.

Although most UTI pathogens in geriatric patients are similar to those found in healthy individuals, the prevalence of multidrug-resistant (MDR) organisms has been rising, particularly in cases requiring hospitalization, making treatment more complex [3]. *Escherichia coli* (*E. coli*) remains the most commonly identified pathogen; however, there has been a notable increase in the prevalence of bacteria producing extended-spectrum beta-lactamases (ESBL) in recent years. These resistant organisms contribute to treatment failures, complications, and prolonged hospital stays [4]. The spread of resistant microorganisms is particularly driven by factors such as invasive procedures, prolonged use of antibiotics, and the hospital environment [5].

Among the key risk factors influencing the development and progression of UTIs are advanced age, comorbid conditions (such as diabetes mellitus, cardiovascular diseases, and renal disorders), immunosuppressive therapy, and the use of invasive medical devices [6,7]. In older adults, the convergence of these factors significantly increases vulnerability to infections. In particular, invasive procedures such as urinary catheterization serve as major triggers in the pathogenesis of UTIs. In catheterized patients, infections are often associated with more resistant pathogens, complicating treatment and adversely affecting overall health status [8].

One of the major challenges in the treatment of UTIs in recent years has been the evolving antibiotic resistance patterns of causative microorganisms. Notably, common pathogens such as *E. coli*, *Klebsiella pneumoniae*, and *Enterococcus* spp. have increasingly demonstrated resistance to modern antibiotics [9,10,11]. The growing prevalence of multidrug-resistant (MDR) bacteria limits therapeutic options and contributes to treatment failures. Moreover, ESBL-producing and carbapenem-resistant organisms pose a significant threat in hospitalized UTI cases [12]. Infections caused by these resistant strains not only complicate management but also prolong hospital stays and elevate in-hospital mortality rates. Another critical factor influencing UTI prognosis in geriatric patients is their overall clinical condition. Age, comorbidities, and the response to treatment are directly associated with mortality risk. Complications such as septic shock, respiratory failure, and multiple organ dysfunction syndrome can markedly worsen the clinical course in this population. These factors further complicate the treatment process and negatively impact survival outcomes.

This multicenter study was conducted to identify the causative microorganisms in hospitalized elderly patients with UTIs, analyze their antibiotic resistance patterns, and evaluate the risk factors associated with in-hospital mortality. The findings aim to contribute to the development of a more targeted and effective management approach for geriatric patients.

## 2. Results

A total of 776 geriatric patients from six tertiary hospitals, four urban settlements from the Aegean Region (Izmir, Manisa, Eskisehir, Turkey), and two rural settlements from the Central Anatolia Region (Kayseri, Niğde, Turkey) were included in the study. Of the patients participating in the study, five were from a public hospital and one was from a private hospital. The median age of the study population was 78.90 ± 7.65 years. Of these, 353 (45.5%) were female and 423 (54.5%) were male. While 656 patients (84.5%) were monitored in regular wards, 120 (15.5%) required intensive care unit (ICU) admission. Upon presentation, 299 patients (38.5%) reported dysuria, 52 (6.7%) had hematuria, 82 (10.6%) experienced flank pain, 125 (16.1%) had groin pain, 290 (37.4%) had urinary incontinence, 159 (20.5%) presented with nausea, 115 (14.8%) with vomiting, 342 (44.1%) had fever, 198 (25.5%) experienced altered mental status, and 324 (41.8%) had poor oral intake. Baseline characteristics and presenting complaints are summarized in Table 1.

Regarding comorbidities, 557 patients (71.8%) had hypertension, 318 (41%) had diabetes mellitus, 188 (24.2%) had chronic obstructive pulmonary disease (COPD), 424 (54.6%) had coronary artery disease (CAD), 339 (43.7%) had dementia, 159 (20.5%) had cerebrovascular disease (CVD), 108 (13.9%) had congestive heart failure (CHF), 277 (35.7%) had chronic kidney disease (CKD), and 251 (32.3%) had benign prostatic hyperplasia (BPH). Additionally, 3 patients (0.4%) had undergone renal transplantation, 73 (9.4%) had a history of kidney stones, and urogenital malignancy was present in 106 patients (13.7%). Chronic indwelling catheter use was recorded in 98 patients (12.6%), nephrostomy in 41 (5.3%), and JJ stent in 30 (3.9%). A summary of comorbidities and risk factors is presented in Table 2.

Urine cultures were positive in 605 patients (78%), while 171 (22%) had negative cultures. Among culture-positive patients, 556 (91.9%) had Gram-negative bacteria and 49 (8.1%) had Gram-positive bacteria as causative agents. *E. coli* was the most commonly isolated pathogen among Gram-negative organisms, detected in 360 cases (64.7%). Other pathogens included *Klebsiella* spp. in 123 cases (22.1%), *Pseudomonas* spp. in 39 (5.9%), *Proteus* spp. in 13 (2.3%), and *Acinetobacter* spp. in 5 cases (0.9%). Other organisms were isolated in 22 patients (4%). Antibiotic resistance analysis of Gram-negative isolates showed that 1.2% of *E. coli* isolates were resistant to carbapenems, and 56.8% were ESBL producers. Among *Klebsiella* spp., 37.8% were resistant to carbapenems and 65.1% were ESBL producers. The resistance profiles of Gram-negative pathogens are presented in Table 3.

Analysis of the predictive factors for ESBL production showed significant associations with urogenital cancer (*p* = 0.009), the presence of urinary tract foreign bodies (*p* = 0.026), hospitalization within the past 6 months (*p* < 0.001), recent antibiotic use within the last 3 months (*p* < 0.001), and the type of Gram-negative pathogen (*p* < 0.001). In the binary logistic regression analysis, independent predictors of ESBL production were urogenital malignancy (HR: 2.30, CI: 1.17–4.51, *p* = 0.016) and antibiotic use within the past 3 months (HR: 3.16, CI: 1.99–5.02, *p* < 0.001). These associations are detailed in Table 4. Regarding carbapenem resistance, statistically significant associations were observed with kidney stone presence (*p* = 0.031), urogenital malignancy (*p* = 0.001), the presence of urinary tract foreign bodies (*p* < 0.001), hospitalization in the previous 6 months (*p* < 0.001), recent antibiotic use (*p* < 0.001), and pathogen type (*p* < 0.001). In the multivariate analysis, independent predictors of carbapenem resistance were the absence of kidney stones (HR: 0.08, CI: 0.01–0.65, *p* = 0.018), hospitalization within the last 6 months (HR: 8.55, CI: 3.14–23.24, *p* < 0.001), and infection with *non-E. coli* Gram-negative bacteria. These results are shown in Table 5.

Among the patients included in the study, 676 (87.1%) were discharged, while 100 (12.9%) died during hospitalization. In the univariate analysis, several factors showed a significant association with increased mortality risk. Binary logistic regression identified ICU admission at presentation, infection with a Gram-positive organism, the presence of COPD or urinary incontinence, and altered mental status at presentation as independent predictors of in-hospital mortality. The associations between clinical presentations, comorbidities, and mortality risk are presented in Table 6.

## 3. Discussions

In this study, the etiology, antimicrobial resistance patterns, and factors associated with mortality in hospitalized geriatric patients with UTIs were investigated. The findings provide valuable insights from both microbiological and clinical perspectives.

Despite advances in modern medicine, UTIs remain a significant global public health issue, affecting millions and contributing to hundreds of thousands of deaths annually [13]. Advanced age, female sex, and the presence of comorbidities are known to increase the incidence and clinical complexity of UTIs in older adults. Gram-negative bacteria are the most common causative agents, accounting for approximately 80–85% of all cases [2]. In the elderly, UTIs may present across a broad clinical spectrum ranging from asymptomatic bacteriuria to UTI-associated septic shock and account for 15–30% of all infections in this age group. Prevalence increases with age, affecting about 10% of women over the age of 65 and up to 30% of women over 85 years [14]. Treatment has become increasingly complicated due to rising antibiotic resistance, particularly among *E. coli* and *Klebsiella* spp., the predominant members of the *Enterobacterales* family [15]. In line with previous studies, *E. coli* and *Klebsiella* spp. were also the most frequently isolated pathogens in our study, confirming their leading role in UTIs among the elderly [4,9]. Although the distribution of causative agents can vary geographically, *Enterobacterales* species are consistently predominant. Early identification of likely pathogens is critical for the selection of appropriate empirical antimicrobial therapy before culture results become available.

In elderly patients, several factors associated with advanced age contribute to both increased susceptibility and higher morbidity and mortality. These include malnutrition, immunosenescence (age-related decline in immune function), poorly controlled diabetes mellitus, urinary retention or incontinence due to impaired bladder control, constipation, prolonged hospitalizations, vaginal atrophy, prostatic hyperplasia, suboptimal hygiene, and altered mental status [1,16]. Our study also highlights the high prevalence of comorbid conditions in this patient population. While typical UTI symptoms include fever, dysuria, urgency, flank pain, burning sensations, suprapubic tenderness, and intermittent urination, the clinical presentation in elderly patients can differ markedly [6,17]. In this age group, UTIs may manifest atypically, often without fever, and instead present as delirium, confusion, dizziness, lethargy, falls, urinary incontinence, or anorexia. These atypical symptoms can obscure the clinical picture, particularly when patients are unable to clearly articulate urinary symptoms, thereby complicating the diagnostic process [2,16,18]. Awareness of such presentations is essential to prevent delays in diagnosis and treatment.

The increasing prevalence of CRE is alarming, as it poses a growing life-threatening public health issue. The rise in CRE rates over the years, coupled with the lack of effective alternative therapies and the high costs of treatment, has reached a critical level [19,20,21]. Countries with stringent antibiotic stewardship programs have managed to maintain relatively stable resistance rates. For example, a study from the Netherlands monitoring women with urinary tract infections between 2004 and 2014 showed an increase in *E. coli* strains producing resistance enzymes such as ESBLs, but no significant rise in overall antibiotic resistance was observed [22]. In contrast, a European surveillance study conducted between 2014 and 2019, involving approximately 700,000 isolates from urinary tract infections, reported increasing antibiotic resistance, including rare but notable instances of resistance to carbapenems and colistin [23,24]. In our study, similarly, ESBL production was observed in approximately 56% of *E. coli* isolates, while carbapenem resistance remained low at around 1%. Following *E. coli*, *Klebsiella* spp. were the second most frequently isolated pathogens in UTIs, though their prevalence varies across studies [25,26]. The growing carbapenem resistance in *Klebsiella* infections poses a significant threat to public health. Inappropriate or excessive antibiotic use—often resulting from misdiagnosis, particularly in elderly patients—is believed to play a crucial role in this trend [27,28]. In 2019, the prevalence of multidrug resistance in *Klebsiella* spp. was approximately 15%, increasing to 20–30% by 2022 [29,30]. In our study, this rate exceeded 35%, which may be attributed to the elderly nature of our study population, in whom frequent and/or inappropriate antibiotic use is more common.

Numerous studies have investigated the predictive factors for ESBL production and carbapenem resistance. Liu et al. identified advanced age, prolonged hospitalization, recent urological invasive procedures, and antibiotic use within the past three months as factors associated with ESBL production [31]. Another study found that previous antibiotic treatment, a low Winston severity score (≤2), and healthcare-associated acquisition were significantly linked to ESBL-producing infections [32]. Similarly, a meta-analysis involving 5597 patients identified prior antibiotic use, previous hospital admissions, and a history of UTIs as significant predictors of ESBL production [33]. In line with these findings, our study also demonstrated that the presence of urogenital malignancy and antibiotic use within the past three months were independently associated with ESBL-producing infections. Several studies have also focused on factors contributing to carbapenem resistance. Rima et al. reported that cerebrovascular disease, a history of hematopoietic stem cell transplantation, the presence of chronic wounds, and the use of meropenem within three months prior to infection were significantly associated with *CRE* [34]. In another recent study, a Charlson Comorbidity Index (CCI) ≥3, central venous catheterization, hospitalization within the previous three months, and prior exposure to carbapenems or broad-spectrum β-lactams were identified as independent risk factors for *carbapenem-resistant Klebsiella pneumoniae (CRKP)* infection [35]. In our study, recent hospitalization within the past six months, a history of nephrolithiasis, and infections caused by *non-E. coli* pathogens—particularly *Klebsiella* spp.—were found to be independent predictors of carbapenem resistance. Considering the previous data in the literature and the results of our study, it should be kept in mind that antibiotic resistance may be present, especially in patients with urological malignancy, recurrent hospital admissions, urological interventions, or a history of stones. If a narrow-spectrum antibiotic is to be started, the patient should be closely monitored, and if no antibiotic response is obtained, a broader-spectrum antibiotic should be preferred without delay.

Previous studies have shown that infections caused by resistant organisms are associated with increased mortality [36]. In our study, mortality was significantly higher among patients who required ICU admission at presentation, those with altered mental status, those infected with Gram-positive pathogens, and those with underlying urinary incontinence or COPD. However, we did not observe a direct association between antimicrobial resistance and mortality. This may be attributable to the timely and appropriate initiation of empirical antibiotic therapy. Although the distribution of UTI pathogens shows consistency across age groups and geographical regions, antimicrobial resistance rates vary widely. Therefore, it is essential for clinicians to be familiar with local resistance patterns to guide the selection of appropriate empirical treatments.

The strength of our study lies in its multicenter design, involving patients from diverse geographical regions, which enhances the generalizability of the findings at a national level. Additionally, as all participants were aged 65 years and older, the study provides valuable insights specific to the geriatric population. However, the retrospective design and the variability in patient populations across different centers constitute important limitations. Another important limitation of our study is that the transmission of the resistance gene was not analyzed using molecular techniques (e.g., PCR, sequencing).

## 4. Materials and Methods

This study was designed as a retrospective, multicenter investigation and included patients who were hospitalized with a diagnosis of urinary tract infection. Inclusion criteria were the following: age 65 years or older; presentation to the emergency department or outpatient clinic between 1 January 2019 and 30 August 2024; and a confirmed diagnosis of urinary tract infection.

The diagnosis of UTI was based on a combination of clinical and laboratory findings, as outlined below by the following: Clinical criteria: The presence of UTI-related symptoms such as fever (≥38 °C), dysuria, hematuria, flank pain, groin pain, urinary incontinence, nausea, vomiting, altered mental status, and impaired oral intake. Microbiological criteria: Biochemical and complete blood count (CBC) parameters obtained at presentation were evaluated. Urinalysis and direct microbiological examination included leukocyte count, hematuria, and nitrite positivity. Urine culture results, including identified microorganisms and their antibiotic susceptibility profiles, were analyzed. Concurrent blood culture results were reviewed to determine the presence of bacteremia.

Exclusion criteria were the following: incomplete medical records, patients managed exclusively in an outpatient setting (i.e., not hospitalized), and fewer than 10 leukocytes per high-power field (hpf) on initial urinalysis.

During the diagnostic process, patients’ medical history, physical examination findings, and clinical–laboratory results were evaluated collectively. Comorbidities (e.g., diabetes mellitus, chronic kidney disease), medical history, administered treatment protocols, and total duration of antibiotic use were recorded. Previous applications, medication, and disease reports of the patients included in the study were reviewed through the hospital electronic record system and comorbid diseases were recorded. Regardless of whether the patient was admitted to the hospital as an inpatient or outpatient, previous applications were reviewed and the history of antibiotic use was recorded.

Urine samples were collected either as midstream clean-catch specimens under sterile conditions or via catheterization. These samples were inoculated onto appropriate culture media and incubated using standard microbiological techniques. After incubation, the grown microorganisms were identified using conventional methods and/or automated identification systems (e.g., VITEK^®^ 2: a tool for the rapid detection of ESBL production that is based on simultaneous assessment of the inhibitory effects of cefepime, cefotaxime, and ceftazidime, BD Phoenix™, Sparks, MD, USA). Antibiotic susceptibility testing of the isolated microorganisms was performed in accordance with the Clinical and Laboratory Standards Institute (CLSI) guidelines. Susceptibility analyses were carried out using either the disk diffusion method or minimum inhibitory concentration (MIC) testing. Carbapenem resistance was defined as the presence of resistance to at least one carbapenem (meropenem, ertapenem, imipenem). Antibiotic panels were selected based on agents commonly used in the participating centers.

All statistical analyses were conducted using SPSS software (version 30.0, IBM Corp., Armonk, NY, USA). Categorical variables were presented as frequencies and percentages. The association between patients’ presenting clinical complaints, comorbidities, and mortality risk was evaluated using logistic regression analysis. A *p*-value of <0.05 was considered statistically significant. This study was conducted with multicenter ethics committee approval obtained from Niğde Ömer Halisdemir University and was carried out in accordance with the principles of the Declaration of Helsinki.

## 5. Conclusions

Our study showed that the most common etiological agents of urinary tract infections in the geriatric population are *E. coli* and *Klebsiella* species. Moreover, the antimicrobial resistance rates in elderly patients were comparable to those reported in studies that included younger adult populations. These findings are significant as they underscore the consistency of pathogen profiles across age groups. Additionally, the study provides valuable insights into the predictive factors for ESBL production and carbapenem resistance, supported by a relatively large patient cohort, thereby contributing meaningful data to the existing literature.

## Figures and Tables

**Table 1:** Demographic and clinical characteristics along with presenting symptoms of the patients.

Parameter	N (%)
Gender Female Male	353 (45.5) 423 (54.5)
Place of Admission Ward Intensive Care Unit	656 (84.5) 120 (15.5)
Dysuria Present Absent	299 (38.5) 477 (61.5)
Hematuria Present Absent	52 (6.7) 724 (93.3)
Flank Pain Present Absent	82 (10.6) 694 (89.4)
Groin Pain Present Absent	125 (16.1) 651 (83.9)
Urinary Incontinence Present Absent	290 (37.4) 486 (62.6)
Nausea Present Absent	159 (20.5) 617 (79.5)
Vomiting Present Absent	115 (14.8) 661 (85.2)
Fever Present Absent	342 (44.1) 434 (55.9)
Confusion Present Absent	198 (25.5) 578 (74.5)
Poor Oral Intake Present Absent	324 (41.8) 452 (58.2)

**Table 2:** Comorbidities of the patients included in the study.

Comorbidities	N (%)
Hypertension Present Absent	557 (71.8) 219 (28.2)
Diabetes Mellitus Present Absent	318 (41) 458 (59)
Chronic Obstructive Pulmonary Disease Present Absent	188 (24.2) 588 (75.8)
Coronary Artery Disease Present Absent	424 (54.6) 352 (45.4)
Dementia Present Absent	339 (43.7) 437 (56.3)
Cerebrovascular Disease Present Absent	159 (20.5) 617 (79.5)
Congestive Heart Failure Present Absent	108 (13.9) 668 (86.1)
Chronic Kidney Disease Present Absent	277 (35.7) 499 (64.3)
Benign Prostatic Hyperplasia Present Absent	251 (32.3) 525 (67.7)
Renal Transplant Present Absent	3 (0.4) 773 (99.6)
Kidney Stone Present Absent	73 (9.4) 703 (90.6)
Urogenital Malignancy Present Absent	106 (13.7) 670 (86.3)
Urinary Foreign Body Chronic Catheter Use Present Absent Nephrostomy Present Absent Double-J Stent Present Absent	98 (12.6) 678 (87.4) 41(5.3) 735 (94.7) 30 (3.9) 746 (3.9)

**Table 3:** Relationship between carbapenem resistance and ESBL production in patients infected with Gram-negative pathogens.

	Carbapenem		Extended-Spectrum Beta-Lactamase		Son Durum	
	Sensitive *n* (%)	Resistance *n* (%)	Nonproducer *n* (%)	Producer *n* (%)	Alive *n* (%)	Dead *n* (%)
*Escherichia coli*	332 (98.8)	4 (1.2)	111 (43.2)	146 (56.8)	313 (86.9)	47 (13.1)
*Klebsiella*	74 (62.2)	45 (37.8)	30 (34.9)	56 (65.1)	110 (89.4)	13 (10.6)
*Pseudomonas*	26 (83.9)	5 (16.1)	2 (20)	8 (80)	30 (90.9)	3 (9.1)
Other	18 (94.7)	1 (5.3)	8 (57.1)	6 (42.9)	19 (86.4)	3 (13.6)
*Proteus*	11 (91.7)	1 (8.3)	3 (42.9)	4 (57.1)	10 (76.9)	3 (23.1)
*Acinetobacter*	2 (50)	2 (50)	0	2 (100)	4 (80)	1 (20)

**Table 4:** Relationship between ESBL production and clinical parameters.

	ESBL Production Pearson Chi-Square Analysis			Binary Logistic Regression Analysis		
	No	Yes	*p* Score	Odds Ratio	Confidence Interval	*p* Score
Gender Female Male	83 (48.0) 71 (34.5)	90 (52.0) 135 (65.5)	0.008	Ref. 1.44	0.90–2.29	0.124
Age 65–74.9 75–84.9 ≥85	57 (35.8) 59 (42.4) 38 (40.6)	102 (64.2) 80 (57.6) 43 (59.4)	0.221			
Place of Admission Ward Intensive Care Unit	132 (40.6) 22 (40.7)	193 (59.4) 32 (59.3)	0.986			
Diabetes Mellitus Absent Present	82 (37.4) 72 (45.0)	137 (62.6) 88 (55.0)	0.139			
Benign Prostatic Hyperplasia Absent Present	117 (39.9) 37 (43.0)	176 (60.1) 49 (57.0)	0.608			
Kidney Stone Absent Present	142 (41.5) 12 (32.4)	200 (58.5) 25 (67.6)	0.285			
Urogenital Malignancy Absent Present	137 (43.6) 17 (26.2)	177 (56.4) 48 (73.8)	0.009	Ref. 2.30	1.17–4.51	0.016
Urinary Foreign Body Absent Present	125 (43.9) 29 (30.9)	160 (56.1) 65 (69.1)	0.026	Ref. 1.08	0.60–1.94	0.788
Hospitalization Within 6 Months Absent Present	98 (52.7) 56 (29.0)	88 (47.3) 137 (71.0)	<0.001	Ref. 1.40	0.81–2.40	0.219
Antibiotic Use Within 3 Months Absent Present	75 (60.5) 79 (31.0)	49 (39.5) 176 (69.0)	<0.001	Ref. 3.16	1.99–5.02	<0.001
Agent *Escherichia coli* *Klebsiella* *Pseudomonas* *Proteus* *Acinetobacter*	111 (43.2) 30 (34.9) 2 (20.0) 3 (42.9) 0 (0)	146 (56.8) 56 (65.1) 8 (80.0) 4 (57.1) 2 (100.0)	<0.001	Ref. 0.97 2.73 0.69 -	0.56–1.70 0.51–14.52 0.13–3.60 -	0.803 0.938 0.239 0.669 0.999

**Table 5:** Relationship between carbapenem resistance and clinical parameters.

	Carbapenem Resistance Pearson Chi-Square Analysis			Binary Logistic Regression Analysis		
	No	Yes	*p* Score	Odds Ratio	Confidence Interval	*p* Score
Gender Female Male	225 (90.7) 244 (87.1)	23 (9.3) 36 (12.9)	0.192			
Age 65–74.9 75–84.9 ≥85	168 (89.4) 187 (90.3) 114 (85.7)	20 (10.6) 20 (9.7) 19 (14.3)	0.401			
Place of Admission Ward Intensive Care Unit	388 (88.4) 81 (91.0)	51 (11.6) 8 (9.0)	0.473			
Diabetes Mellitus Absent Present	274 (87.8) 195 (12.2)	38 (90.3) 21 (9.7)	0.378			
Benign Prostatic Hyperplasia Absent Present	337 (88.5) 132 (89.8)	44 (11.5) 15 (10.2)	0.660			
Kidney Stone Absent Present	421 (41.5) 48 (32.4)	58 (58.5) 1 (67.6)	0.031	Ref. 0.082	0.01–0.65	0.018
Urogenital Malignancy Absent Present	408 (90.7) 61 (78.2)	42 (9.3) 17 (21.8)	0.001	Ref. 0.75	0.33–1.66	0.484
Urinary Foreign Body Absent Present	375 (91.5) 94 (79.7)	35 (8.5) 24 (20.3)	<0.001	Ref. 1.16	0.53–2.55	0.701
Hospitalization Within 6 Months Absent Present	243 (98.0) 226 (80.7)	5 (2.0) 54 (19.3)	<0.001	Ref. 8.55	3.14–23.24	<0.001
Antibiotic Use Within 3 Months Absent Present	158 (96.9) 311 (85.2)	5 (3.1) 54 (14.8)	<0.001	Ref. 1.44	0.40–5.18	0.571
Agent *Escherichia coli* *Klebsiella* *Pseudomonas* *Proteus* *Acinetobacter*	332 (98.8) 74 (62.2) 26 (83.9) 11 (91.7) 2 (50.0)	4 (1.2) 45 (37.8) 5 (16.1) 1 (8.3) 2 (50.0)	<0.001	1 44.76 18.13 4.68 61.99	15.28–131.07 4.34–75.75 0.47–3.46.64 5.89–652.32	<0.001 <0.001 <0.001 0.188 0.001

**Table 6:** Relationship between patients’ clinical presenting complaints, comorbidities, and risk of mortality (binary logistic regression analysis).

	Odds Ratio	Confidence Interval	*p* Score
Place of Admission Intensive Care Unit vs. ward	4.35	2.44–7.75	<0.001
Agent Gram-Positive vs. Negative	4.09	1.83–9.14	0.001
COPD Present vs. Absent	2.32	1.31–4.10	0.004
Urinary Incontinence Present vs. Absent	1.88	1.09–3.24	0.022
Altered Mental Status Present vs. Absent	3.39	1.93–5.96	<0.001
Poor Oral Intake Present vs. Absent	1.56	0.91–2.69	0.104
Coronary Artery Disease Present vs. Absent	1.23	0.70–2.16	0.470
Chronic Heart Failure Present vs. Absent	1.05	0.52–2.10	0.882
Dementia Present vs. Absent	0.85	0.44–1.64	0.640

## Data Availability

The data that support the findings of this study are available from the corresponding author, [Çiğdem Yıldırım], upon reasonable request.
